# Physician Compensation Arrangements and Financial Performance Incentives in US Health Systems

**DOI:** 10.1001/jamahealthforum.2021.4634

**Published:** 2022-01-28

**Authors:** Rachel O. Reid, Ashlyn K. Tom, Rachel M. Ross, Erin L. Duffy, Cheryl L. Damberg

**Affiliations:** 1 RAND Corporation, Santa Monica, California and Boston, Massachusetts; 2 Division of General Internal Medicine and Primary Care, Brigham and Women’s Hospital, Boston, Massachusetts; 3 Harvard Medical School, Boston, Massachusetts

## Abstract

**Importance:**

Public and private payers continue to expand use of alternative payment models, aiming to use value-based payment to affect the care delivery of their contracted health system partners. In parallel, health systems and their employment of physicians continue to grow. However, the degree to which health system physician compensation reflects an orientation toward value, rather than volume, is unknown.

**Objective:**

To characterize primary care physician (PCP) and specialist compensation arrangements among US health system–affiliated physician organizations (POs) and measure the portion of total physician compensation based on quality and cost performance.

**Design, Setting, and Participants:**

This study was a cross-sectional mixed-methods analysis of in-depth multimodal data (compensation document review, interviews with 40 PO leaders, and surveys conducted between November 2017 and July 2019) from 31 POs affiliated with 22 purposefully selected health systems in 4 states. Data were analyzed from June 2019 to September 2020.

**Main Outcomes and Measures:**

The frequency of PCP and specialist compensation types and the percentage of compensation when included, including base compensation incentives, quality and cost performance incentives, and other financial incentives. The top 3 actions physicians could take to increase their compensation. The association between POs’ percentage of revenue from fee-for-service and their physicians’ volume-based compensation percentage.

**Results:**

Volume-based compensation was the most common base compensation incentive component for PCPs (26 POs [83.9%]; mean, 68.2% of compensation; median, 81.4%; range, 5.0%-100.0% when included) and specialists (29 POs [93.3%]; mean, 73.7% of compensation; median, 90.5%; range, 2.5%-100.0% when included). While quality and cost performance incentives were common (included by 83.9%-56.7% of POs for PCPs and specialists, respectively), the percentage of compensation based on quality and cost performance was modest (mean, 9.0% [median, 8.3%; range, 1.0%-25.0%] for PCPs and 5.3% [median, 4.5%; range, 0.5%-16.0%] for specialists when included). Increasing the volume of services was the most commonly cited action for physicians to increase compensation, reported as the top action by 22 POs (70.0%) for PCPs and specialists. We observed a very weak, nonsignificant association between the percentage of revenue of POs from fee for service and the PCP and specialist volume-based compensation percentage (*r* = 0.08; *P* = .78 and *r* = −0.04; *P* = .89, respectively).

**Conclusions and Relevance:**

The results of this cross-sectional study suggest that PCPs and specialists despite receiving value-based reimbursement incentives from payers, the compensation of health system PCPs and specialists was dominated by volume-based incentives designed to maximize health systems revenue.

## Key Points

**Question:**

Do health system physician compensation arrangements primarily incentivize volume or value?

**Findings:**

This cross-sectional mixed-methods study of 31 physician organizations affiliated with 22 US health systems found that volume was a component of primary care and specialist compensation for most POs (83.9% and 93.3%, respectively), representing a substantial portion of compensation when included (mean, 68.2% and 73.7%, respectively). While most primary care and specialist compensation arrangements included performance-based incentives, they averaged less than 10% of compensation.

**Meaning:**

The study results suggest that despite growth in value-based payment arrangements from payers, health systems currently incentivize physicians to maximize volume, thereby maximizing health system revenues.

## Teaser

This cross-sectional study examines primary care physician and specialist compensation arrangements among US health system–affiliated physician organizations and the portion of total physician compensation based on quality and cost performance.

## Introduction

Since passage of the US Affordable Care Act, public and private payers in the US have undertaken various payment reforms to improve quality and reduce spending. Alternative payment models (APMs) and value-based payment (VBP) seek to redirect the health system’s focus toward producing value instead of volume.^1,2,3,4,5,6,7,8^ Whether it is a response to value-oriented payment models or not,^9,10,11^ during the same period, health systems and their employment of physicians have grown.^12,13,14,15,16^

A payment hierarchy exists in the US health care system. Reimbursement mechanisms used by payers, such as fee for service, capitation, or APMs, create incentives for health systems and POs. In turn, these organizations create incentives for physicians through compensation packages, which may or may not reflect the same structure and incentives as those that POs face from payers. Evidence suggests that POs are selective in which incentives they pass along to physicians.^3,4^ Conversely, others have observed that even when employed physicians are salaried, their compensation and incentives often incorporate elements of their practice’s payment environment, with salaried physicians in heavily capitated environments rewarded based on net revenue and salaried physicians in heavily fee-for-service environments rewarded based on volume (ie, production of services or productivity).^17^ In surveys, most employed physicians reported salary-based compensation, but volume-based and mixed (ie, base salary plus other financial incentives) are also common.^18^ However, salaries themselves may be based on prior volume, panel size, or other factors that may not be well captured by survey-based data collection.

Compensation and financial incentives are a lever for health systems to affect the care delivery of physicians. Given increased exposure of health systems to APMs, it is important to understand the degree to which health system compensation and incentives for physicians reflect the same value-based incentives provided by payers. Further, there is limited information on physician compensation variation among health systems and physician types (ie, primary care physicians [PCPs] vs specialists). To our knowledge, no prior study of physician compensation and incentives has specifically focused on health systems. In this study, we used in-depth multimodal data collection to examine variation in PCP and specialist compensation and incentives among a purposive sample of health system POs.

## Methods

### RAND Health System Study

This study was a component of the larger RAND Health System Study, in which in-depth interviews were conducted with senior leaders among a purposive sample of health systems in 4 states (California, Minnesota, Wisconsin, and Washington) that were selected because of their advanced collection and public reporting of PO performance data through health care measurement and improvement collaboratives and because they represented diverse market characteristics.^19,20^ Among health systems that publicly reported performance data in state health care measurement and improvement collaboratives, a purposive sample of 24 were selected for the RAND Health System Study to achieve variability on key attributes (eg, size and performance). The incentives component collected information on the PCP compensation of 31 POs and specialist compensation of 30 POs within 22 of those health systems between November 2017 and July 2019 and analyzed data from July 2019 to September 2020. The in-depth multimodal data collection included semistructured phone interviews, review of compensation and performance metric documentation, and a structured survey. The RAND Corporation's institutional review board approved this study; oral informed consent was obtained from PO leaders during the initial phone interview. This study conforms to the Standards for Reporting Qualitative Research (SRQR) and Strengthening the Reporting of Observational Studies in Epidemiology (STROBE) reporting guidelines.

### Data Collection for Incentive Substudy

We carefully screened to identify participants from each PO who were knowledgeable about physician compensation and incentives. All interviews were led by a female physician researcher with an MD or a policy researcher with a PhD. An initial phone interview was conducted to determine compensation structure and the percentage of total compensation of each of the following elements represented for PCPs and specialists: base compensation incentives (ie, salary, capitation, volume, and profit-sharing), performance-based incentives (ie, clinical quality or patient safety, panel size, patient satisfaction/experience, efficient utilization of resources, total cost of care, and access), and other incentives (ie, hierarchical condition category/risk adjustment factor coding, code submission or accuracy, and citizenship or participation in PO requirements or activities). Leaders of POs could indicate use of more than 1 base compensation incentive category, and base compensation incentives could vary in relative contribution (ie, be the primary component or a marginal incentive). Leaders of POs provided documents that described their physician compensation structures and the measures that were incentivized. The POs then completed a structured survey that addressed the use of incentives in the following categories: volume, panel size, clinical quality of care, patient satisfaction/experience, efficient utilization of resources, access, total cost of care, and hierarchical condition category/risk adjustment factor coding (eMethods in the Supplement). It also addressed the top 3 actions physicians could take to increase compensation. We conducted a follow-up interview after review of the compensation documents and survey responses to confirm our understanding of the PO’s physician compensation structure and the percentage of total compensation that each element represented. During these interviews, we elicited details about how the compensation package components were derived. For example, if salaries were determined using the volume of services from a prior year, we categorized that compensation as volume based. The interview also addressed how the PO was reimbursed by payers (ie, percentage of revenue from fee for service, capitation, or another source). Interview-derived PO revenue information was cross-referenced with responses from another survey of selected POs from the RAND Health System Study when available (ie, percentage of fee-for-service, capitation, global payment, bundled payment, or other from of payment). Participants could reference documents and materials when responding to surveys and interview questions; additional clarification, if needed, was provided via follow-up emails or calls. Interviews were recorded and transcribed. Physician compensation and incentives and PO revenue data were maintained and processed in Microsoft Excel; all study team members reviewed data and arrived at consensus regarding interpretation thereof.

### Data Analysis

We present the frequency of different types of compensation for PCPs and specialists. We also assessed the mean, median, and range of the percentage of compensation that a given category represented for PCPs and specialists when included. We summarized the frequencies of the top 3 actions PCPs and specialists could take to increase their compensation. Lastly, to assess the degree to which the reimbursement from payers that health systems received translated into physician compensation, we computed the Pearson correlation coefficient between the percentage of a PO’s revenue from fee for service and their PCP and specialist volume-based compensation percentage among POs that reported revenue information. Physician compensation and incentives and PO revenue data were maintained and processed in Microsoft Excel and analyzed in Microsoft Excel and SAS, version 9.4 (SAS Institute).

## Results

This study included 40 PO leader participants (30 men [75%] and 10 women [25%]) from 31 POs in 22 health systems, of which all 31 were nonprofit, 14 (45.2%) were academic affiliated, 27 (87.1%) were medical groups, and 4 (12.9%) were independent practice associations. Geographically, 15 (48.4%) were in California, 3 (9.7%) in Washington, 7 (22.6%) in Minnesota, and 6 (19.4%) in Wisconsin (Table 1). All 31 POs provided information about PCP compensation models, 30 (96.8%) provided information about specialist compensation models, and 15 (48.4%) provided information about overall revenue streams.

**Table 1.** Physician Organization Characteristics

Characteristic	No. (%)
Health systems, No.	22
Physician organizations, No.	31
Physician organization characteristics	
Organization type	
Medical group	27 (87.1)
Independent practice association	4 (12.9)
Location	
California	15 (48.4)
Minnesota	7 (22.6)
Washington	3 (9.7)
Wisconsin	6 (19.4)
Nonprofit	31 (100)
Academic medical center affiliated	14 (45.2)

Regarding base compensation incentives, volume of services was the most commonly included compensation mechanism for PCPs (26 of the 31 PCP compensation models [83.9%]) and specialists (28 of the 30 specialist compensation models [93.3%]) (Table 2). Among the 26 POs that included volume as a component their PCP compensation model, it comprised an average of 68.2% of the total compensation for PCPs (median, 81.4%; range, 5.0%-100%). Among the 28 POs that included volume as a component their specialist compensation model, it comprised an average of 73.7% of the total compensation for specialists (median, 90.5%; range, 2.5%-100%). Capitation and salary were also commonly included base compensation mechanisms for PCPs (9 POs [29.0%] and 8 POs [26%], respectively) and specialists (3 POs [10%] and 8 POs [27%], respectively). Among POs that incorporated these components in PCP compensation, capitation comprised an average of 33.3% of total compensation among those 9 POs (median, 27.0%; range, 3.8%-90.0%), and salary comprised an average of 69.7% of total compensation among those 8 POs (median, 80.0%; range, 15.8%-89.0%). For the 3 POs that incorporated capitation in specialist compensation, it comprised an average of 54.7% of total compensation (median, 52.0%; range, 13.0%-99.0%), and for the 8 POs that incorporated salary in specialist compensation, it comprised an average of 67.9% of total compensation (median, 77.5%; range, 38.0%-92.5%).

**Table 2.** Compensation Types for PCPs and Specialists

Financial incentive type	PCPs				Specialists			
	POs including, No. (%)	Compensation when included, %			POs including, No. (%)	Compensation when included, %		
		Mean	Median	Range		Mean	Median	Range
Base compensation incentives	31 (100)	86.3	85.0	62.0-100	30 (100)	93.1	95.0	65.0-100
Salary	8 (25.8)	69.7	80.0	15.8-89.0	8 (26.7)	67.9	77.5	38.0-92.5
Capitation	9 (29.0)	33.3	27.0	3.8-90.0	3 (10.0)	54.7	52.0	13.0-99.0
Volume	26 (83.9)	68.2	81.4	5.0-100	28 (93.3)	73.7	90.5	2.5-100.0
Profit sharing	4 (12.9)	11.6	12.6	6.0-15.3	2 (6.7)	10.1	10.1	10.0-10.2
Quality and cost performance incentives	26 (83.9)	9.0	8.3	1.0-25.0	17 (56.7)	5.3	4.5	0.5-16.0
Clinical quality or patient safety	21 (67.7)	4.7	4.8	0.8-13.7	12 (40.0)	3.2	3.0	1.1-8.0
Panel size	8 (25.8)	4.8	4.8	0.2-11.0	1 (3.3)	5.0	5.0	5.0
Patient satisfaction/experience	16 (51.6)	2.8	2.5	0.9-6.0	15 (50.0)	2.1	2.0	0.5-3.6
Efficient utilization of resources	6 (19.4)	4.4	3.8	1.0-10.0	2 (6.7)	2.0	2.0	0.5-3.6
Total cost of care	1 (3.2)	5.0	5.0	5.0	0	NA	NA	NA
Access	7 (22.6)	1.5	1.0	0.1-5.0	2 (6.7)	7.5	7.5	2.0-13.0
Other incentives	16 (51.6)	7.6	5.1	1.5-20.0	9 (30.0)	6.6	5.0	0.5-27.0
HCC/RAF coding	6 (19.4)	5.7	5.8	1.0-10.0	0	NA	NA	NA
Code submission or accuracy	2 (6.5)	7.8	7.8	3.5-12.0	1 (3.3)	4.5	4.5	4.5
Citizenship or participation	13 (41.9)	4.0	5.0	1.2-7.3	11 (36.7)	5.6	3.0	0.5-27.0
Other	16 (51.6)	5.4	5.0	1.0-15.0	9 (30.0)	5.2	3.0	1.0-15.0

Abbreviations: HCC, hierarchical condition category; NA, not applicable; PCPs, primary care physicians; POs, physician organizations; RAF, risk adjustment factor.

For PCPs, 26 of the 31 PO compensation models (83.9%) included quality and cost performance-based financial incentives, averaging 9.0% of total compensation (median, 8.3%; range, 1.0%-25.0%) when included. The most commonly incentivized areas for PCPs were clinical quality or patient safety (21 POs [67.7%]) and patient experience or satisfaction (16 POs [51.6%]). Among POs that incentivized these categories, incentives averaged 4.7% (median, 4.8%; range, 0.8%-13.7%) and 2.8% (median, 2.5%; range, 0.9%-6.0%) of total compensation when included, respectively. Among other incentives for PCPs, citizenship or participation incentives were the categories most commonly included (13 of 31 POs [41.9%]) and averaged 4.0% of compensation (median, 5.0%; range, 1.2%-7.3%) when included.

In contrast, fewer specialist compensation models (17 of 30 POs [56.7%]) included performance-based financial incentives, averaging 5.3% of total compensation (median, 4.5%; range, 0.5%-16.0%) when included. As with PCPs, the most commonly included incentives for specialists focused on performance for clinical quality or patient safety (12 of 30 POs [40.0%]) and patient experience or satisfaction (15 of 30 POs [50.0%]). Among POs that incentivized these categories, incentives averaged 3.2% (range, 1.1%-8.0%) and 2.1% (median, 2.0%; range, 0.5%-3.6%) of total compensation when included, respectively. Citizenship or participation incentives were the other specialist incentives most commonly included (11 of 30 POs [36.7%]) and averaged 5.6% of compensation when included (median, 3.0%; range, 0.5%-27.0%).

**Figure 1.**
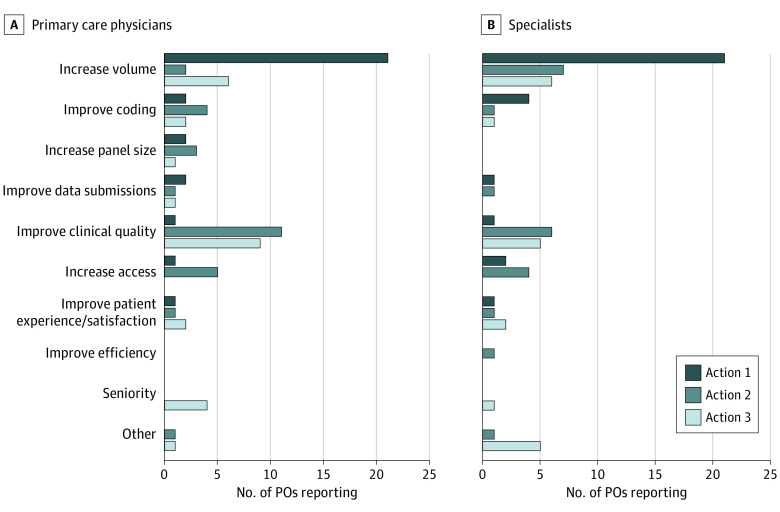
Top 3 Actions Physicians Can Take to Increase Compensation POs indicates physician organizations.

Increasing volume of services was the most common action PO leaders reported physicians could take to increase their compensation, which was noted as the top action by 21 of the 30 POs that completed surveys (70.0%) for PCPs and specialists and within the top 3 actions 29 times among 84 total actions for PCPs (34.5%) and 34 times among the 72 total actions for specialists (47.2%) (Figure 1). Improving clinical quality was the next most commonly cited way to increase physician compensation, noted 19 times among the top 3 actions for PCPs by POs (22.6%) and 12 times among the top 3 actions for specialists (16.7%).

**Figure 2.**
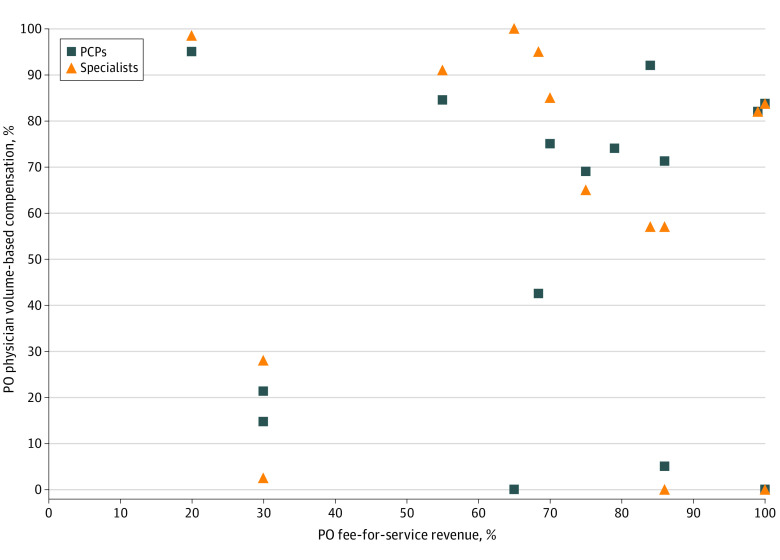
Association Between Physician Organization (PO) Fee-for-Service Revenue and Volume-Based Compensation for PO Physicians PCP indicates primary care physician.

Among the 15 POs that also reported data on external financial incentives from payers, in comparing their percentage of revenue derived from the fee-for-service payments of payers with the percentage of their physician compensation that was volume-based, we found a very weak correlation for PCPs (*r* = 0.08; *P* = .78) and specialists (*r* = −0.04; *P* = .89) (Figure 2). Most, but not all, POs had reported that a higher percentage of specialist compensation was based on volume. The POs with the highest proportion of their revenue from fee for service reported an equal percentage of compensation based on volume for PCPs and specialists, although the actual reported percentage of volume-based compensation varied widely among these primarily fee-for-service–reimbursed POs.

## Discussion

This in-depth multimodal cross-sectional assessment of compensation and incentives among health system–affiliated POs for which there is greater exposure to VBP and APM arrangements compared with independent practices found that volume was the most common form of base compensation by a wide margin, being included by more than 80% and 90% of POs for PCPs and specialists, respectively, and representing more than two-thirds of compensation when included. Similarly, actions to increase volume were the most commonly cited means for physicians to increase their compensation. Base compensation incentives for physicians were not dominated by population or value-oriented payments, with only a third of POs reporting inclusion of capitation with PCPs and averaging only about a third of total compensation when included. Performance-based financial incentives for value-oriented goals, such as clinical quality, cost, patient experience, and access, were commonly included in compensation but represented a small fraction of total compensation for PCPs and specialists in health systems, operating at the margins to affect physician behavior. Taken together, these findings suggest that despite growth in APMs and VBP arrangements, these value-based incentives were not commonly translated into health system physician compensation, which was dominated by volume-oriented incentives.

The dominance of volume-based incentives among health system physician compensation is consistent with prior surveys that reflected the remuneration of physicians, including employed physicians and medical groups, more broadly.^3,18,21,22,23^ Even physicians in practices who received significant shares of their revenue from capitation still often receive some volume-based incentives at the margins.^3^ The study findings of a very weak association between the percentage of PO revenue from fee for service and the percentage of PCP and specialist compensation based on volume contrasts somewhat with prior research that found that the percentage of PO revenue from health management organizations or capitation or managed care penetration in the market was negatively associated with physician volume-based compensation.^24,25^ Health system POs may be translating external APM or VBP incentives into aligned financial incentives more for PCPs than for specialists, with most reporting a higher proportion of volume-based compensation for specialists than PCPs. This difference for PCPs and specialists may be partially traceable to the relative number of well-validated clinical quality measures available for some specialist care. Additionally, financial incentives for physicians are only 1 tool that POs and health systems have to affect care delivery and respond to APMs and VBP incentives; organizations may use many other approaches (eg, nonfinancial physician incentives, ordering and referral support and guidance, practice and organizational supports, and leadership incentives) to affect care. Nonetheless, the modest size of these quality and cost performance incentives for PCPs and specialists compared with the base compensation incentives suggest that their potential to change behavior is likely to be marginal. This is underscored by health system PO leaders citing actions to increase volume of services as by far the most common mechanism for physicians to increase their compensation.

Practice leaders have traced the prominence of volume incentives in individual physician compensation to the familiarity of physicians with the mechanism as well as ongoing links to extant fee-for-service-payment structures.^4^ This study found less compensation via salary than prior surveys of employed physicians.^18,21,22^ This could be because of the study’s focus on health system–affiliated POs, but may also be attributable to the multimodal data collection with a combination of survey, document review, and interviews, which allowed us to unpack compensation more comprehensively and understand any volume-based or other incentives that underlie salaries. Despite rapid growth in APMs and VBP arrangements, fee-for-service reimbursement from payers is common, both as a payment arrangement in and of itself and as a chassis on which APMs and VBP arrangements have been built.^8,16,26,27,28^ As arrangements like global payments and direct contracting gain additional ground, potentially shifting more POs reimbursement away from fee for service, it remains to be seen whether evolution in physician compensation will occur.

The findings of frequent but modest incentive compensation for health system PCPs and specialists for clinical quality, patient experience, access, and other areas correspond with prior findings in various settings, both before and after adoption of the Affordable Care Act, with and without participation in accountable care organizations.^22,24,29,30^ It is challenging to translate risk-bearing payment arrangements and many measures of quality, utilization, or value to the individual physician level for payment purposes owing to limitations in panel sizes and reliability concerns with measuring individual physician performance.^31,32,33,34,35^ The increasing intricacy of individual APM finical incentives, including risk-bearing arrangements, coupled with the cumulative complexity of incentives across payers, has been cited as a rationale for practices and POs to serve as a buffer between payers’ incentives and physicians.^3,4^ This purposeful disconnect between the incentives and financial risk that POs face from payers and those passed on in physician compensation likely also contributes to the dominance of volume-based compensation and modesty of quality and cost performance incentives.^36^ While pay-for-performance incentives serve as a mechanism for POs to signal priorities and affect behavior and have been a focus in the literature, base compensation incentives may ultimately affect physician behavior more. Owing to the relative size of financial incentives and overall compensation package complexity, actions to increase volume were the most commonly cited as a means for PCPs and specialists to increase their compensation. Increasing the frequency and relative size of salary or capitation base compensation incentives might result in differential prioritization of the actions of physicians to increase compensation and therefore affect care delivery and value.

### Limitations

This study has limitations. Our findings from a purposive sample of health system–affiliated POs in 4 states may not generalize to unaffiliated POs in other regions of the country where market characteristics or payment model penetration may vary. However, these states were selected to represent variation in the national marketplace. For example, California had a higher presence of capitation, Washington had more limited VBP and APM exposure, and Minnesota had a relatively consolidated market. Further, the health systems selected represent a diversity of size and performance. This purposive sample enabled our in-depth multimodal data collection approach, facilitating a more comprehensive understanding of compensation and incentives than broad survey-only methods. All included health systems were nonprofit, as are most health systems nationally (97.0% nonprofit or government owned)^37^; for-profit health systems might be expected to have compensation models that greater emphasize volume. A substantial minority of included POs were academic affiliated, which was similar to health systems nationally (37.2% affiliated with a major teaching hospital)^37^; the association between the volume-based compensation for physicians and fee-for-service revenue may vary for academic systems. The data collection focused on PO leaders rather than physicians; PO leaders have a global view of incentives that their organizations receive from payers and compensation and incentives for physicians across their PO, but the perceptions of physicians on what is affecting their compensation may differ. Finally, our analysis of the association between PO fee-for-service revenue and volume-based physician compensation was based on a smaller subsample.

## Conclusions

Despite growth in VBP arrangements and a push to improve value in health care, physician compensation arrangements in health systems do not currently emphasize value. Volume-based incentives dominate health system PCP and specialist compensation, with quality and cost performance incentives representing a relatively small portion of compensation. Many factors may limit alignment of value-based compensation for physicians, and the best mix of incentives to optimize value-based care delivery is unknown. However, as health systems and their employment of physicians continue to grow, greater translation of the value-over-volume incentives of payers into physician compensation may be necessary to realize the full potential of value-oriented payment reform.

## Notes

Supplement. eMethods.
