# Supplementary material for: Physician Compensation Arrangements and Financial Performance Incentives in US Health Systems
Source: JAMA Health Forum. 2022 Jan 28;3(1):e214634. doi: 10.1001/jamahealthforum.2021.4634 (PMC8903115; doi:10.1001/jamahealthforum.2021.4634)
Supplement: Supplement. — eMethods. [file jamahealthforum-e214634-s001.pdf]

Supplemental Online Content

Reid RO, Tom AK, Ross RM, Duffy EL, Damberg CL. Physician compensation arrangements and financial performance incentives in US health systems. *JAMA Heath Forum*. 2022;3(1):e214634. doi:10.1001/jamahealthforum.2021.4634

eMethods.

This supplemental material has been provided by the authors to give readers additional information about their work.

© 2022 Reid RO et al. *JAMA Health Forum*.

eMethods.

SELECTED QUESTIONS FROM THE HEALTH SYSTEM PHYSICIAN ORGANIZATION LEADER SURVEY INSTRUMENT

1. Does your physician organization measure, report, and/or provide financial incentives to **primary care physicians** on their individual performance for any of the following performance areas?
   - **"Measure"** means your organization ***tracks*** physician performance in this area.
   - **"Report"** means your organization provides ***scorecards or other types of performance reports*** to physicians.
   - **"Financial incentive"** means your organization includes the performance area in its ***variable compensation*** for physicians

Performance Area (Primary Care Physicians)	Measure	Report	Provide Financial Incentive	Do Not Measure, Report, or Financially Incentivize
**Productivity** (e.g., patient visits, charges, encounter data submissions, or RVUs (FFS))	☐	☐	☐	☐
**Panel size** (e.g., number of patients in a panel)	☐	☐	☐	☐
**Clinical quality of care** (e.g., HEDIS, Medicare Star measures)	☐	☐	☐	☐
**Patient experience or satisfaction** (e.g., CAHPS)	☐	☐	☐	☐
**Efficient utilization of resources** (e.g., reducing inpatient days)	☐	☐	☐	☐
**Access** (e.g., accepting new patients, extended hours)	☐	☐	☐	☐
**Total cost of care** (e.g., shared savings payments)	☐	☐	☐	☐
**HCC/RAF coding***	☐	☐	☐	☐
**Other** (if yes, please specify): ______________________________	☐	☐	☐	☐

*HCC=Hierarchical Condition Category. RAF=Risk Adjustment Factor. Used to adjust Medicare payments

© 2022 Reid RO et al. *JAMA Health Forum*.

2. Does your physician organization measure, report, and/or provide financial incentives to some or all **specialty care physicians** on their individual performance for any of the following performance areas?
    - **"Measure"** means your organization ***tracks*** physician performance in this area.
    - **"Report"** means your organization provides ***scorecards or other types of performance reports*** to physicians.
    - **"Financial incentive"** means your organization includes the performance area in ***variable compensation*** for physicians.

Performance Area (Specialty Care Physicians)	Measure	Report	Provide Financial Incentive	Do Not Measure, Report, or Financially Incentivize
**Productivity** (e.g., patient visits, charges, encounter data submissions, or RVUs (FFS))	☐	☐	☐	☐
**Panel size** (e.g., number of patients in a panel)	☐	☐	☐	☐
**Clinical quality of care** (e.g., HEDIS, Medicare Star measures)	☐	☐	☐	☐
**Patient experience or satisfaction** (e.g., CAHPS)	☐	☐	☐	☐
**Efficient utilization of resources** (e.g., reducing inpatient days)	☐	☐	☐	☐
**Access** (e.g., accepting new patients, extended hours)	☐	☐	☐	☐
**Total cost of care** (e.g., shared savings payments)	☐	☐	☐	☐
**HCC/RAF coding**	☐	☐	☐	☐
**Other** (if yes, please specify): ________________________	☐	☐	☐	☐

*HCC=Hierarchical Condition Category. RAF=Risk Adjustment Factor. Used to adjust Medicare payments

© 2022 Reid RO et al. *JAMA Health Forum*.

3. For a typical **primary care physician** in your organization, what are the three top easiest actions he/she could do to increase his/her compensation? *List them in order, with Action 1 being the easiest.*

Action—Primary Care Physicians
**Action #1**
**Action #2**
**Action #3**

4. For a typical **specialty care physician** in your organization, what are the three top easiest actions he/she could do to increase his/her compensation? *List them in order, with Action 1 being the easiest.*

Action—Specialty Physicians
**Action #1**
**Action #2**
**Action #3**

© 2022 Reid RO et al. *JAMA Health Forum*.

SELECTED QUESTIONS FROM HEALTH SYSTEM PHYSICIAN ORGANIZATION LEADER SEMI-STRUCTURED INTERVIEW GUIDE

1. **How are your primary care physicians paid?** *(meaning: Is their compensation primarily based on salary, capitation, or something else?)*

2. **For a typical primary care physician in your organization, how much of their compensation falls into the following categories?** *(probe: do family medicine physicians and internal medicine physicians have same compensation approach?)*

Base salary	____%
Capitation	____%
Fee-for-service (e.g., RVUs, productivity)	____%
Performance-based incentives (e.g., bonuses or penalties based on quality, patient experience, utilization of resources, etc.)	____%
Profit sharing	____%
Other (if yes, please specify): ____	____%
**TOTAL (should add to 100%)**	

3. **How are your specialty care physicians paid?** (meaning Is their compensation primarily based on salary, capitation, or something else?)

4. **For a typical specialty care physician in your organization, how much of their compensation falls into the following categories?**

Base salary	____%
Capitation	____%
Fee-for-service (e.g., RVUs, productivity)	____%
Performance-based incentives (e.g., bonuses or penalties based on quality, patient experience, utilization of resources, etc.)	____%
Profit sharing	____%
Other (if yes, please specify): ____	____%
**TOTAL (should add to 100%)**	

5. **What share of your physician organization's total revenues do you estimate come from the following sources?** (*interviewer: should add to 100%)*

   Fee-for-service ___________________

   Capitation ________________________

   Other sources _____________________

© 2022 Reid RO et al. *JAMA Health Forum.*
